# Unsafe disposal of feces of children <3 years among households with latrine access in rural Bangladesh: Association with household characteristics, fly presence and child diarrhea

**DOI:** 10.1371/journal.pone.0195218

**Published:** 2018-04-05

**Authors:** Mahfuza Islam, Ayse Ercumen, Sania Ashraf, Mahbubur Rahman, Abul K. Shoab, Stephen P. Luby, Leanne Unicomb

**Affiliations:** 1 Environmental Intervention Unit, Enteric and Respiratory Infections Program, Infectious Disease Division, International Centre for Diarrheal Disease Research, Bangladesh (icddr,b), Dhaka, Bangladesh; 2 University of California, Berkeley, CA, United States of America; 3 Department of International Health, Johns Hopkins Bloomberg School of Public Health, Baltimore, MD, United States of America; 4 Woods Institute for the Environment, Stanford University, Stanford, United States of America; Boston University, UNITED STATES

## Abstract

**Background:**

Young children frequently defecate in the living environment in low-income countries. Unsafe child feces disposal has been associated with risk of diarrhea. Additionally, reported practices can underestimate socially undesirable unhygienic behaviors. This analysis aimed to assess (1) the sensitivity of reported child feces disposal practices as an indicator for observed presence of human feces in the domestic environment, (2) household characteristics associated with reported unsafe feces disposal and (3) whether unsafe feces disposal is associated with fly presence and diarrhea among children <3 years.

**Methods:**

We recorded caregiver-reported feces disposal practices for children <3 years; unsafe disposal was defined as feces put/rinsed into a drain, ditch, bush or garbage heap or left on the ground and safe disposal as feces put/rinsed into latrine or specific pit or buried. We conducted spot checks for human feces, counted flies in the compound and recorded caregiver-reported child diarrhea prevalence among 803 rural Bangladeshi households. We assessed associations using generalized estimating equations (GEE) and generalized linear models (GLM) with robust standard errors.

**Results:**

Unsafe disposal of child feces was reported by 80% of households. Reported disposal practices had high sensitivity (91%) but low positive predictive value (15%) as an indicator of observed feces in the compound. Unsafe disposal was more common among households that reported daily adult open defecation (PR: 1.13, 1.02–1.24) and had children defecating in a nappy or on the ground versus in a potty (PR: 2.92, 1.98–4.32), and less common in households where adults reported always defecating in latrines (PR: 0.91, 0.84–0.98). The presence of observed human feces was similarly associated with these household characteristics. Reported unsafe feces disposal or observed human feces were not associated with fly detection or child diarrhea.

**Conclusion:**

Despite access to on-site sanitation, unsafe child feces disposal was reported by the majority of households. However, this practices was not associated with diarrhea; suggesting that child feces may not be the most important fecal exposure. Before resources are invested to improve child feces management practices, studies should explore whether these contribute meaningfully to risk of enteric disease.

## Introduction

One billion people worldwide (18% of the global population) practice open defecation and in low-income countries nine out of the ten residents who defecate in the open live in rural areas [1]. The WHO/UNICEF Joint Monitoring Programme (JMP) for Water Supply and Sanitation estimates that 610 million people in South Central Asia defecate in the open [2]. In Bangladesh, reported open defecation decreased from 42% in 2003 to 1% in 2015, measured as access to a toilet [2]; the nationally representative Bangladesh National Hygiene Baseline Survey, conducted in 2013, 2% of households had no access to a toilet and 4% lacked access in rural areas [3]. However, these figure based on latrine access could underestimate the actual practice of open defecation. Even in settings where there is wide spread latrine coverage and adult open defecation is infrequent, young children continue to defecate directly in the living environment [4]. Safe disposal practices of child feces include disposing of them in a latrine or burying them [5]. Unsafe child feces disposal practices include disposing of child feces in open areas or not disposing of them at all. While Bangladesh has a very high rate of latrine access (96%), it has the second lowest levels of reported safe disposal of child feces in the South Central Asia region [4]. This could be because child potties and diapers are rarely used in Bangladesh [6]. Children typically defecate directly on the ground in Bangladesh [7, 8], as found in other countries [9–13] and child feces are then swept into bushes near the household [14].

Open feces in the compound can increase risk of fecal exposure for compound members, especially young children who spend time in the courtyard area and have hand contact with the feces or with soil that has been contaminated by feces [15]. Child feces in the domestic environment can also provide breeding sites for flies, which are known vehicles of diarrheal pathogen transmission [16]. Unsafe disposal of child feces has been associated with increased the risk of diarrheal disease in some settings [10, 17] as well as markers of environmental enteropathy and impaired child growth [18]. A study in Bangladesh found that disposal of child feces into improved latrines was associated with a decreased risk of helminthiasis by 35% in children <2 years [19]. However, a recent meta-analysis found an association between unsafe child feces disposal and child diarrhea in only two out of five studies reviewed [20].

Sanitation programs have limited impact on child feces disposal practices [20, 21]. Factors leading to unsafe disposal of child feces despite latrine access have not yet been well explored. Insights into household or caregiver characteristics associated with unsafe child feces disposal can inform promotion efforts to reduce the potential risk of diarrheal disease from exposure to environmental fecal contamination from child feces. Additionally, child feces management practices are typically recorded by caregiver report. Self-reported practices can be subject to courtesy bias that might underestimate true levels by underreporting socially undesirable behaviors. Self-reported child feces management practices have not been validated with objective measurements such as spot check observations. This study, among households with latrine access in rural Bangladesh, aimed to: (1) determine household characteristics associated with reported unsafe disposal of young children’s feces and observed human feces within the compound, (2) determine the sensitivity, specificity, positive predictive value (PPV) and negative predictive value (NPV) of reported child feces disposal practices compared to observed presence of human feces in the domestic environment, and (3) assess the association between unsafe child feces disposal and (i) fly presence and (ii) diarrhea among children <3 years living in the household.

## Methods

### Study setting and design

We conducted a cross-sectional analysis nested within the baseline data collection for a large cluster-randomized controlled trial of water, sanitation, hygiene and nutrition interventions in central rural Bangladesh ([22]; www.washbenefits.net). The WASH Benefits trial enrolled 5551 households with pregnant women in their first or second trimester. In our analysis, we included data from WASH Benefits households that had a latrine in their compound, had at least one child <3 years old and reported a feces disposal event (i.e., defecation by the child in a location other than latrine, thus requiring caregiver handling) within the last two days, yielding 803 households for analysis.

### Data collection

Field workers visited households between May 2012 and July 2013. Field staff used a structured questionnaire to record reported defecation and feces disposal practices for children <3 years. We defined unsafe child feces disposals as feces put/rinsed into a drain, ditch, bush or garbage heap or left on the ground, and safe disposal as feces put/rinsed into latrine or specific pit or buried. Field staff recorded demographic information, and the caregiver-reported 7-day prevalence of diarrhea (≥3 loose stools within 24 hours), presence of blood in stool and negative control outcomes (skin rashes, bruises/scrapes) among children <3 years. Field workers also conducted spot checks in each household to observe the sanitation facilities and record the presence of any human feces within the courtyard and compound areas. In a random subset of households (N = 107), the field team set up three 1.5-footstrips of unbaited sticky fly tape at three locations (latrine, food preparation and waste disposal areas) and counted the number of flies captured after 24 hours. The combined number of flies from all three locations was used in the analyses.

We categorized household sanitation access using the WHO/UNICEF Joint Monitoring Programme (JMP) definitions for improved and unimproved latrines [1]. As an alternative categorization, we classified latrines that effectively isolate feces from the environment with an intact water seal as “hygienic” latrines. This included flush latrines connected to a piped sewer system or septic tank, pit latrines with a functional water seal and composting latrines. If the latrines failed to effectively separate feces from the environment we classified them as “unhygienic” latrines. This included flush latrines connected to a canal or ditch, pit latrines with no or broken water seals, and hanging latrines. This definition did not consider shared usage; JMP defines all shared latrines as unhygienic.

### Statistical analysis

We conducted bivariate and multivariable analysis with modified Poisson regression for all binary outcome using generalized estimating equations (GEE) and negative binomial regression for the fly count outcome using generalized linear models (GLM) both with robust standard errors to account for the clustered nature of WASH Benefits households. We assessed the associations between reported unsafe child feces disposal and (1) household and caregiver characteristics, (2) the probability of fly detection and the number of flies detected, and (3) the prevalence of gastrointestinal and negative control symptoms in children <3 years. We repeated these analyses using observed presence of human feces (instead of reported disposal practices) as a robustness check against reporting bias. For each outcome we investigated, we identified potential confounders as factors that were predictive of the dependent variable and also likely to affect the independent variables of interest. We identified household wealth as a potential confounding factor and used principal component analysis to calculate a household wealth index using assets and housing materials [23, 24]. This index was used as a covariate to control for household wealth. In multivariable models we included all covariates that were associated with the dependent variable at the p<0.2 level in bivariate analyses.

To assess the potential for reporting bias of child feces disposal practices, we calculated the sensitivity, specificity, PPV and NPV of self-reported feces disposal practice against observed feces in the compound area. We conducted all statistical analyses using STATA software (version 13).

### Ethical considerations

All households provided written informed consent. The protocol was reviewed and approved by human subjects review committees at the International Centre for Diarrheal Disease Research, Bangladesh (icddr,b) and the University of California, Berkeley.

## Results

**Fig 1:**
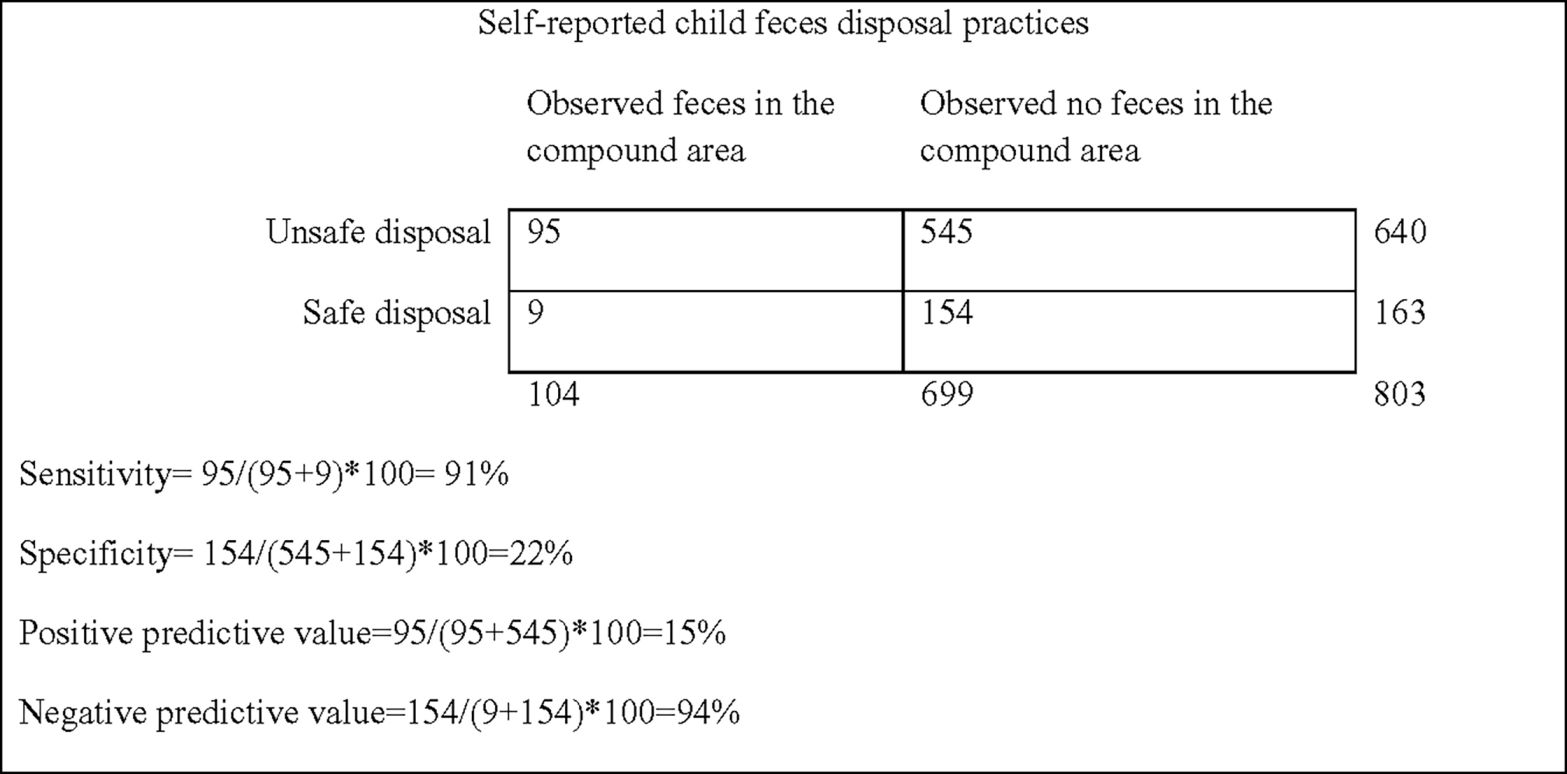
Two by two tables illustrating sensitivity, specificity, positive predictive value and negative predictive value of self-reported child feces disposal practice, and observed feces in the compound area.

Among the 803 households with access to a latrine and at least one child <3 years, 80% (n = 640) reported unsafe feces disposal for the last defecation event for children <3 years. Among these, 64% (n = 408) reported disposing feces in the bush, 18% (n = 117) in open waste heaps, 13% (n = 85) in drains, and 11% (n = 68) left the feces on the ground. Multiple disposal locations were reported by some households. Among the 640 households reporting unsafe child feces disposal, 5% (n = 35) had observed feces in the courtyard and 15% (n = 95) within the compound, indicating a PPV of 15% for reported feces disposal as an indicator of observable feces in the compound. Conversely, among the 104 households with observable feces in the compound area, 91% (n = 95) reported unsafe child feces disposal, indicating a sensitivity of 91%. Reported feces disposal had a specificity of 22% and an NPV of 94% against observable feces in the compound (Fig 1).

Among the 107 households selected for fly density assessment, flies were captured in 94% (n = 101) of households. The mean number of flies captured after 24 hours was 29 (SD = 20) and the median was 27 (range: 4–86). The caregiver-reported 7-day prevalence of diarrhea in children <3 years was 6% and the 7-day prevalence of blood in stool was 1.3%.

In bivariate analyses, the prevalence of caregiver-reported unsafe child feces disposal was significantly higher among households that had children ≤18 months old, households where the child <3 years old was reported to defecate in a nappy or on the ground versus in a potty, households where daily defecation in the open was reported for adults, and households with an unimproved, unhygienic or shared latrine (Table 1). Unsafe child feces disposal was less commonly reported by households where the child’s mother was <20 years old. Mothers’ age was correlated with their level of education; mothers aged ≤20 years had on average 6.78 (SD = 2.82) years of education, while those aged 20–25 years had 5.73 (SD = 3.24) years, those aged 25–30 years had 4.94 (SD = 3.61) years and those aged >30 years had 3.15 (SD = 3.78) years of education (ANOVA p<0.001). Unsafe child feces disposal was less commonly reported by households where adults reported consistent latrine use for defecation, and a potty was available for child defecation (Table 1).

**Table 1:** Factors associated with reported unsafe disposal of feces of children <3 years and observed human feces within the compound among households with latrine access.

Characteristics		Reported unsafe feces disposal				Observed human feces within the compound			
			Unsafe disposal^a^	Bivariate	Multivariable		Feces observed	Bivariate	Multivariable
		N	n (%)	PR^b^ (95% CI)	PR^c^ (95% CI)	N	n (%)	PR^b^ (95% CI)	PR^c^ (95% CI)
**Socioeconomics and demographics**									
Age of the child									
	≤18 months old	192	156 (81)	**1.19 (1.11, 1.28)**	**1.07 (1.02, 1.13)**	163	19 (12)	0.92 (0.57, 1.46)	1.01 (0.63, 1.61)
	>18 months and <36 months old	611	414 (68)	ref		674	86 (13)	ref	
Sex of child									
	Male	371	298 (80)	1.02 (0.95, 1.09)	1.01 (0.94, 1.07)	386	56 (15)	1.34 (0.93, 1.91)	1.32 (0.93, 1.89)
	Female	432	342 (79)	ref		451	49 (11)	ref	
Mother’s age									
	≤20 years	159	117 (74)	**0.86 (0.75, 0.97)**	**0.88 (0.78, 0.98)**	165	9 (5.5)	**0.34 (0.14, 0.79)**	**0.42 (0.18, 0.98)**
	21 years to 25 years	381	308 (81)	0.94 (0.85, 1.04)	0.97 (0.89, 1.06)	399	52 (13)	0.79 (0.47, 1.34)	0.96 (0.57, 1.64)
	26 years to 30 years	192	154 (80)	0.93 (0.78, 0.94)	0.91 (0.82, 1.01)	199	31 (16)	0.93 (0.52, 1.66)	1.04 (0.58, 1.87)
	>30 years	71	61 (86)	ref		74	13 (18)	ref	
Mother's education									
	No or primary	438	374 (85)	**1.17 (1.09, 1.26)**	1.06 (0.98, 1.15)	448	76 (17)	**2.21 (1.46, 3.34)**	**1.77 (1.06, 2.96)**
	Secondary or above	365	266 (73)	ref		389	29 (7.5)	ref	
Father's education									
	No or primary	532	447 (84)	**1.18 (1.08, 1.28)**	1.05 (0.95, 1.15)	545	81 (15)	**1.74 (1.15, 2.65)**	1.05 (0.62, 1.75)
	Secondary or above	269	192 (71)	ref		290	24 (8.3)	ref	
Wealth index^d^									
	Low	268	228 (85)	**1.26 (1.14, 1.39)**	**1.19 (1.06, 1.32)**	281	46 (16)	**2.16 (1.33, 3.50)**	1.53 (0.88, 2.67)
	Middle	268	232 (87)	**1.28 (1.17, 1.41)**	**1.23 (1.11, 1.36)**	277	38 (14)	**1.80 (1.10, 2.95)**	1.40 (0.83, 2.37)
	High	267	180 (67)	ref		279	21 (7.5)	ref	
**Reported sanitation behaviors**									
Latrine use by household members for defecation									
	Always used by adults	672	524 (78)	**0.87 (0.81, 0.93)**	**0.90 (0.84, 0.97)**	706	77 (11)	**0.48 (0.32, 0.71)**	**0.62 (0.40, 0.96)**
	Sometimes/never used by adults	118	106 (90)	ref		118	27 (23)	ref	
	Always used by 8 to 15 yrs old children	233	183 (79)	**0.86 (0.76, 0.96)**		242	30 (12)	0.58 (0.28, 1.18)	
	Sometimes/never used by 8–15 yrs old children	37	34 (92)	ref	—^e^	37	8 (22)	ref	—^e^
	Always used by 3-<8 yrs old children	254	194 (76)	**0.86 (0.79, 0.93)**		165	18 (11)	**0.49 (0.30, 0.82)**	
	Sometimes/never used by 3-<8yrs old children	207	184 (89)	ref	—^e^	192	42 (22)	ref	—^e^
Open defecation by household members									
	Daily by adults	3	3 (100)	**1.25 (1.21, 1.30)**	**1.11 (1.02, 1.20)**	3	2 (67)	**5.45 (2.39, 12.41)**	**2.80 (1.37, 5.73)**
	Sometimes/never by adults	800	637 (80)	ref	—^e^	834	103 (12)	ref	—^e^
	Daily by 8–15 yrs old children	18	17 (94)	**1.19 (1.05, 1.35)**		18	4 (22)	1.68 (0.66, 4.28)	
	Sometimes/never by 8–15 yrs old children	256	203 (79)	ref	—^e^	265	35 (13)	ref	—^e^
	Daily by 3-<8 yrs old children	118	109 (92)	**1.17 (1.07, 1.27)**		117	28 (24)	**1.85 (1.20, 2.86)**	
	Sometimes/never by 3-<8 yrs old children	228	180 (79)	ref		239	31 (13)	ref	
Child defecation location (<3 years old)									
	In cloth diaper or ground	741	624 (84)	**3.25 (2.19, 4.83)**	**2.92 (1.98, 4.32)**	737	101 (14)	**3.28 (1.23, 8.77)**	**1.67 (1.11, 2.61)**
	In potty	62	16 (26)	ref		98	4 (4.1)	ref	
Latrine sharing									
	Shared by multiple households	482	402 (83)	**1.12 (1.04, 1.21)**	1.03 (0.95, 1.11)	493	75 (15)	**1.78 (1.18, 2.69)**	1.28 (0.80, 2.04)
	Used by single households	321	238 (74)	ref		344	30 (8.7)	ref	
**Observed sanitation infrastructure**									
Latrine type^f^									
	Unimproved latrine	179	152 (85)	**1.09 (1.01, 1.17)**	1.01 (0.94, 1.09)	181	30 (17)	**1.50 (1.01, 2.23)**	1.12 (0.73, 1.72)
	Improved latrine	624	488 (78)	ref		656	75 (11)	ref	
Latrine characteristic									
	Unhygienic latrine^g^^,^ ^h^	529	440 (83)	**1.14 (1.05, 1.24)**	1.03 (0.95, 1.23)	542	80 (15)	**1.79 (1.18, 2.72)**	1.18 (0.71, 1.94)
	Hygienic latrine	274	200 (73)	ref		295	25 (8.5)	ref	
Potty for child defecation									
	Present	138	79 (57)	**0.68 (0.59, 0.78)**	1.07 (0.95, 1.20)	142	12 (8.5)	0.67 (0.38, 1.16)	1.58 (0.86, 2.91)
	Absent	665	561 (84)	ref		695	93 (13)	ref	
Dedicated tool to clean up feces around household									
	Present	641	510 (79)	0.99 (0.91, 1.08)	0.97 (0.90, 1.06)	664	79 (12)	0.80 (0.52, 1.23)	0.86 (0.57, 1.30)
	Absent	162	130 (80)	ref		173	26 (15)	ref	

PR: Prevalence ratio; CI: Confidence interval

^a^**Unsafe child feces disposal**s defined as feces put/rinsed into drain or ditch/bush or jungle/garbage or left on the ground.

^b^We estimated the prevalence ratio by using generalized estimating equation (GEE) to adjust for clustering.

^c^Multivariable model includes all variables associated with unsafe disposal/feces in the compound in bivariate analyses at p<0.2 level.

^d^**Wealth index** calculated as tertiles from principal component analysis of household assets.

^e^We excluded this variable from the multivariable model because too few households had a child in this age range for robust analysis.

^f^Defined using WHO/UNICEF Joint Monitoring Programme definition for improved and unimproved latrine.

^g^**Unhygienic latrines** include pit latrines with no water seal and absence of lid or pit with no slab or a hanging latrine or latrines directly open to the environment

^h^We excluded shared latrine variable from the multivariable model because of co-linearity with unhygienic latrine.

In multivariable models controlling for wealth and mothers’ education, unsafe feces disposal remained significantly more common in households that had a child ≤18 months old (PR: 1.07, 1.02–1.13), reported daily adult open defecation (PR: 1.13, 1.02–1.24), and allowed children to defecate onto a nappy or on the ground (PR: 2.92, 1.98–4.32). Unsafe feces disposal was less frequently reported in households with mothers aged <20 years (PR: 0.88, 0.78–0.98) and adults reporting that they always use latrines for defecation (PR: 0.91, 0.84–0.98) (Table 1). These household characteristics and practices showed similar associations with observed human feces within the compound (Table 1).

Reported unsafe feces disposal was not significantly associated with the probability of detecting any flies in the compound, the mean number of captured flies (Table 2), 7-day prevalence of diarrhea, presence of blood in stool or negative control outcomes (skin rashes and bruising/scrapes/cuts) among children <3 years (Table 3). We also found a similar lack of associations between these outcomes and observed feces presence in the compound (Tables 2 and 3). For some associations, the confidence intervals were wide when few households had observed feces, especially for rare exposures such as daily open defecation by adults and should therefore be interpreted with caution. The number of flies captured in households with observed human feces was significantly lower in bivariate analyses, and we did not find any other associations in bivariate or in multivariable analyses (Table 2).

**Table 2:** Association between reported child feces disposal practices or observed human feces within the compound and fly presence and density in the households.

Outcomes	Reported unsafe feces disposal				Observed human feces within the compound			
	Households with unsafe disposal^a^	Households with safe disposal	Bivariate model^b^	Multivariable model^c^	Feces observed	Feces not observed	Bivariate model^b^	Multivariable model^c^
	(N = 85)	(N = 22)	PR (95% CI)	PR (95% CI)	(N = 9)	(N = 103)	PR (95% CI)	PR (95% CI)
Flies present, n (%)	80 (94)	21 (95)	0.99 (0.61, 1.59)	0.84 (0.33, 2.10)	8 (89)	98 (95)	0.93 (0.45, 1.92)	0.92 (0.44, 1.94)
Flies absent, n (%)	5 (6)	1 (5)	ref	ref	1 (11)	5 (5)	ref	ref
Number of flies on the fly tape: Mean (SD)	19 (18)	23 (22)	0.99 (0.99, 1.00)	1 (0.99, 1.01)	7 (4)	26 (22)	**0.87 (0.77, 0.98)**	0.93 (0.70, 1.23)

PR: Prevalence ratio; CI: Confidence interval

^a^**Safe child feces disposal**s defined as feces put/rinsed into latrine or specific pit or buried

^b^We determined the prevalence ratio by using general linear model (GLM) to adjust for clusters

^c^Adjusted for wealth index, mother’s education, unimproved latrine, unhygienic latrine, adults daily defecate in the open, child defecation site

**Table 3:** Association between reported child feces disposal practices or observed human feces within the compound and caregiver-reported 7-day prevalence of diarrhea and negative control outcomes in children <3 years.

Outcomes	Reported unsafe feces disposal						Observed human feces within the compound					
	Households with unsafe disposal		Households with safe disposal		Bivariate RR^a^	Multivariable RR^b^	Feces observed		Feces not observed		Bivariate RR^a^	Multivariable RR^b^
	N	n (%)	N	n (%)	PR (95% CI)	PR (95% CI)	N	n (%)	N	n (%)	PR (95% CI)	PR (95% CI)
Diarrhea ^c^	627	65 (10)	162	15 (9.3)	1.13 (0.66, 1.94)	0.97 (0.82, 1.15)	103	8 (7.8)	719	40 (5.6)	1.34 (0.63, 2.85)	1.07 (0.56, 2.05)
Blood in the stool	621	8 (1.3)	159	3 (1.9)	0.68 (0.18, 2.52)	0.90 (0.66, 1.22)	102	3 (2.9)	710	8 (1.1)	2.63 (0.70, 9.79)	1.90 (0.56, 6.14)
Negative control outcomes												
Skin rash	626	62 (9.9)	162	20 (12)	0.80 (0.50, 1.28)	0.95 (0.84, 1.07)	103	5 (4.9)	719	81 (11.3)	0.43 (0.18, 1.04)	0.47 (0.20, 1.09)
Bruising/scrapes/cuts	625	30 (4.8)	162	5 (3.1)	1.57 (0.64, 3.88)	1.08 (0.94, 1.23)	103	5 (4.9)	717	31 (4.3)	1.13 (0.46, 2.74)	1.02 (0.46, 2.28)

PR: Prevalence ratio; CI: Confidence interval

^a^We determined the prevalence ratio by using generalized estimating equation (GEE) to adjust for clusters

^b^Adjusted for wealth index, mother’s education, adults always used latrine, adults daily defecate in the open, child defecation site, shared latrine, unimproved latrine, potty availability

^c^Diarrhea calculated from WHO definition (> = 3 loose stools/24 hours)

## Discussion

In our study, the majority of households reported unsafe child feces disposal despite having on-site latrine access, suggesting that open defecation by young children in this population is substantially more common than the nationwide estimate of 1% for Bangladesh by the JMP [2], which uses lack of latrine access to define open defecation. This is consistent with other studies, which found unsafe child feces disposal reported by 81% of households with latrine access in a small-scale study in rural India [25] and 67% in Ethiopia [26].

Compared to commonly reported unsafe disposal for feces of children <18 months, feces of older children (18–36 months) were more likely to be disposed of safely. This finding was consistent with another study conducted in rural Bangladesh [14]. This could be explained by the perception that stool of young children is typically considered harmless or less harmful and less disgusting than older children’s feces in South Asia [27]. Feces of young children are smaller, smell less, and contain fewer visible food residues [6, 27]. In contrast, the feces of older children are characterized by bad smell and visual food residues which can make the feces be perceived as more disgusting [6].

A study conducted in Tanzania reported that older caregivers were less likely to practice unsafe disposal [28]. This contrasts with our study where younger mothers were less likely to practice unsafe disposal. We found that younger mothers enrolled in our study had higher educational levels. This could indicate that the younger, more educated mothers are more conscious about disposing of child feces safely and are more likely to understand causes of childhood illness [28], therefore practicing more hygienic behaviors to protect their child from illness.

Surprisingly, the presence of an improved or hygienic latrine, child potty or dedicated tool for feces management was not associated with safe disposal of child feces in multivariable analyses, suggesting that presence/absence of hardware had little impact on hygienic feces management. In contrast, households with better sanitation habits overall were more likely to safely dispose of child feces. Households where adults reported daily open defecation were more likely to unsafely dispose of child feces, consistent with another study in Cambodia [29]. This suggests that adults who prioritize hygienic sanitation practices for themselves behave similarly with their children. Messages that promote cessation of open defecation for adults could therefore have the additional benefit of promoting safe child feces management practices. However, reported daily open defecation among adults was low so associations should be interpreted with caution. Child defecation in potties was strongly associated with safe child feces disposal, consistent with studies conducted in Burkina Faso [30], Peru [31] and rural Nigeria [32]. A study conducted in Cambodia found that caregivers were satisfied with potties as they are easy to use, transport, clean and easy to empty/rinse into the latrine [29]. In addition, potties save caregivers time as they do not need to hold and wait with the child while the child is using the potty and caregivers can engage in other household tasks [29]. However, in rural Bangladesh, potty use is very uncommon [14] as rural parents are not aware about the benefits of using potties or may not know how to train their children to use the potty [33]. Exposing caregivers to the advantages of potties and educating them on how to potty-train their children as part of sanitation programs might lead to improved child feces management.

One strength of our analysis was that previous studies relied solely on reported child feces disposal practices whereas we compared reported disposal practices with observed feces within the compound area. Additionally, when we asked about feces disposal practices, we tried to minimize reporting bias by enquiring about the “last time” the child defecated rather than asking about the usual practice for disposal of child feces, as the latter question has been suggested to be more likely to elicit the socially desirable response [34]. The household characteristics and practices associated with reported disposal practices were similar for the objective observation of feces in the compound, suggesting that these associations were not explained by social desirability bias, that is, caregivers reporting safe disposal also reporting other socially desirable behaviors such as consistent latrine use.

In addition, among the households that had observable feces in the compound area, 91% reported unsafe disposal of feces, indicating that the reported practice was highly sensitive as an indicator of visible feces with no evidence that caregivers were underreporting unsafe disposal practices. However, we note that these data were collected from households that did not receive an intervention and/or promotion efforts to reduce unsafe disposal of child feces. It is possible that in an intervention trial that focuses on improving these practices social desirability bias would lead to underreporting of unsafe practices. Additionally, the specificity and PPV of reported feces disposal practices against observed presence of feces was low (i.e., among households where no feces were observed, only a small subset reported safe disposal and among households where unsafe disposal was reported, only a subset had visible feces in the compound). This is likely because only a small proportion (11%) of respondents among those with unsafe disposal practices that reported that they left feces on the ground, compared to the majority (64%) that discarded feces in the bushes. Field workers only inspected the courtyard and open areas within the compound for feces and did not inspect bushes or waste heaps.

We did not find an association between reported unsafe child feces disposal and fly presence or numbers. Similarly, we did not find an association between observed human feces presence in the compound and fly presence. While households with human feces appeared to have a significantly lower number of flies in bivariate analyses, in multivariable analyses the association was not statistically significant. This could suggest that other risk factors like household waste disposal practices, drainage systems, community level sanitary conditions and animal feces are the primary attractants to flies and, given the abundance of fly food in the environment, child feces may contribute too little to make a difference. It is also possible that our sample size for the flies assessment (n = 107) was too small to provide statistical power to detect associations with feces disposal practices for this outcome. There were also very few households with no flies detected (5 households among those with unsafe disposal and1 household among those with safe disposal); these analyses were therefore driven by sparse data.

The evidence to date on the association between unsafe child feces disposal and child diarrhea is mixed. A study in Indonesia found that unsafe child feces disposal behaviors were associated with an increased risk of diarrheal diseases [6]. Two additional intervention studies in rural Bangladesh found that disposing of child feces in a latrine and no visible feces being present in the household compound were associated with a 27–30% reduction in pediatric diarrhea [7, 35]. In contrast, a recent meta-analysis that assessed the health impact of safe feces disposal found that, out of five studies reviewed, only two found a reduction in diarrhea while the others did not find an association [20]. In our study we also did not find an association between unsafe feces disposal or observed feces presence in the compound and child gastrointestinal illness. Some of these analyses were limited by rare outcomes and exposures; for example, among households with observed feces, there were 8 cases of diarrhea and 3 cases with bloody stool. However, while these analyses with sparse data had wide confidence intervals, the overall lack of associations between feces handling and health outcomes was consistent across all exposure and outcome definitions. One possible explanation could be that, in this setting with on-site sanitation access where adult open defecation is rare, unsafe feces disposal is restricted to children mostly. As there are rarely multiple children in the compound that are young enough to indiscriminately defecate in the compound environment, this could suggest that young children may only be infrequently exposed to feces from other children and more commonly exposed to their own feces. Exposure to one’s own feces is unlikely to present a major health risk as any pathogens shed in a child’s stool would be pathogens that the child is already infected with.

This was an observational study and is therefore subject to confounding. For example, households where child feces are managed unsafely could have other unhygienic practices that would affect child diarrheal outcomes. However, we controlled for a wide range of potential confounding factors to minimize this potential source of bias. Additionally, in the analyses assessing the association between child feces disposal and child illness, we would expect confounding from unmeasured factors such as caregivers’ health awareness to bias the observed associations away, and not towards the null (i.e., we would expect mothers who practice unsafe feces disposal to be less health aware and more likely to have other practices that can lead to increased illness in their children). It is therefore unlikely that confounding explains the lack of association between feces disposal and health outcomes. We also found no association between unsafe child feces disposal and negative control outcomes, suggesting no evidence of reporting bias.

Finally, we measured child feces disposal practices and diarrhea outcomes cross-sectionally during the same household visit. In addition, we recorded how caregivers disposed of feces from the child’s last defecation event (within two days of the interview), while the diarrhea prevalence we recorded spanned the 7-day period before the interview. It is therefore possible that the outcomes occurred before the exposure that we measured and that this reverse ordering biased the observed association between feces disposal practices and child gastrointestinal illness toward a null effect. For example, when a child is ill with diarrhea, the caregiver may be more likely to safely dispose of the feces as she could perceive of them as harmful, whereas the feces of a healthy child may be left on the ground. Future studies should record childhood diarrhea symptoms prospectively after collecting feces management data [36].

Most sanitation programs focus on provision of latrine hardware and promotion of latrine use to reduce open defecation. Moreover, open defecation is often measured as access to a latrine which does not reflect open defecation among young children. It has been previously documented that these sanitation improvements do not affect child defecation and child feces management practices [20, 21]. In our study setting, we found that, despite having access to on-site sanitation, 92% of children <3 years defecated in the open and only 11% of households disposed of child feces safely in the latrine. However, we found no increased illness risk associated with the unsafe disposal or observed presence of child feces in the compound. Our findings suggest that child feces may not be a particularly important fecal exposure for childhood diarrhea. Safe disposal of child feces requires complex behavior change, which may be infeasible for caregivers with competing demands on their time in rural low-income country settings. As we and others have not found an impact of child feces on health, before resources are invested to improve child feces management practices, more studies should investigate whether these contribute meaningfully to risk of enteric disease.
